# Phylogenetic and metabolic diversity of microbial communities performing anaerobic ammonium and methane oxidations under different nitrogen loadings

**DOI:** 10.1038/s43705-023-00246-4

**Published:** 2023-04-25

**Authors:** Jie Li, Tao Liu, Simon J. McIlroy, Gene W. Tyson, Jianhua Guo

**Affiliations:** 1 Australian Centre for Water and Environmental Biotechnology, The University of Queensland, St Lucia, QLD Australia; 2 Centre for Microbiome Research, School of Biomedical Sciences, Queensland University of Technology, Translational Research Institute, Woolloongabba, QLD Australia

**Keywords:** Microbial ecology, Water microbiology, Microbial ecology

## Abstract

The microbial guild coupling anammox and nitrite/nitrate-dependent anaerobic methane oxidation (n-DAMO) is an innovative process to achieve energy-efficient nitrogen removal with the beneficial use of methane in biogas or in anaerobically treated wastewater. Here, metagenomics and metatranscriptomics were used to reveal the microbial ecology of two biofilm systems, which incorporate anammox and n-DAMO for high-level nitrogen removal in low-strength domestic sewage and high-strength sidestream wastewater, respectively. We find that different nitrogen loadings (i.e., 0.1 vs. 1.0 kg N/m^3^/d) lead to different combinations of anammox bacteria and anaerobic methanotrophs (“*Candidatus* Methanoperedens” and “*Candidatus* Methylomirabilis”), which play primary roles for carbon and nitrogen transformations therein. Despite methane being the only exogenous organic carbon supplied, heterotrophic populations (e.g., Verrucomicrobiota and Bacteroidota) co-exist and actively perform partial denitrification or dissimilatory nitrate reduction to ammonium (DNRA), likely using organic intermediates from the breakdown of methane and biomass as carbon sources. More importantly, two novel genomes belonging to “*Ca*. Methylomirabilis” are recovered, while one surprisingly expresses nitrate reductases, which we designate as “*Ca*. Methylomirabilis nitratireducens” representing its inferred capability in performing nitrate-dependent anaerobic methane oxidation. This finding not only suggests a previously neglected possibility of “*Ca*. Methylomirabilis” bacteria in performing methane-dependent nitrate reduction, and also challenges the previous understanding that the methane-dependent complete denitrification from nitrate to dinitrogen gas is carried out by the consortium of bacteria and archaea.

## Introduction

A paradigm shift is occurring to transition wastewater treatment from energy-intensive to energy-neutral. This advancement is generally achieved by enhancing bioenergy recovery in the form of biogas and reducing energy and chemical consumption using the autotrophic anaerobic ammonium oxidation (anammox) process. Despite multifaced benefits [1], one of the most critical issues of anammox process is the nitrate accumulation (NH_4_^+^ + 1.32NO_2_^-^ → 1.02N_2_ + 0.26NO_3_^-^ + 2.03H_2_O), which leads to the maximal nitrogen removal efficiency below 90%, unless external organic carbon is supplied.

As one of the simplest and cheapest organic carbons, methane is abundant in biogas generated from anaerobic sludge digestion and in anaerobically treated wastewater. Thus, using methane produced in wastewater treatment plants (WWTPs) to enhance nitrogen removal by anammox is considered economically feasible and environmentally friendly, imparting additional benefits to mitigate greenhouse gas emissions in wastewater sectors. Recent discoveries of microbial processes capable of performing nitrite/nitrate-dependent anaerobic methane oxidation (n-DAMO) have laid the foundation to achieve this aim [2–4]. Specifically, “*Candidatus* Methylomirabilis oxyfera” (namely n-DAMO bacteria) is able to reduce nitrite with methane as the electron donor (3CH_4_ + 8NO_2_^-^ + 8H^+^ → 3CO_2_ + 4N_2_ + 10H_2_O) [3, 4], while a novel archaeon, “*Candidatus* Methanoperedens nitroreducens” (namely n-DAMO archaea) is capable of reducing nitrate to nitrite with methane as the electron donor (CH_4_ + 4NO_3_^-^ → CO_2_ + 4NO_2_^-^ + 2H_2_O) [2]. Since the discovery, the methane-dependent denitrification from nitrate to dinitrogen gas has been considered to be carried out by the labour union of n-DAMO archaea and n-DAMO bacteria performing the two steps in sequence. If methane produced at a WWTP serves as a carbon source for nitrate and nitrite removal when fed to the anammox reactor, this would support complete nitrogen removal without the importation of other organic carbons.

In recent years, a number of technologies have been developed to couple anammox and n-DAMO species for efficient nitrogen removal from various wastewater streams, e.g., the low-strength domestic sewage [5] and the high-strength sidestream wastewater [6]. Two key advantages are identified for this combination. Firstly, complete nitrogen removal can be achieved because n-DAMO archaea can convert the nitrate generated by anammox bacteria to nitrite, which will be further consumed by anammox bacteria and n-DAMO bacteria. Secondly, both n-DAMO bacteria and anammox bacteria are able to remove nitrite, rendering the coupled systems with more robustness against wastewater dynamics. As shown in previous studies [7], high nitrogen removal efficiency (>95%) could be stably maintained in biofilm systems coupling anammox and n-DAMO microorganisms, even if the nitrite to ammonium molar ratio in wastewater varied from 1.2 to 1.8. These are important features for real applications considering the requirement of operational stability in practice.

While previous studies have mainly focused on the structure of microbial communities using 16S rRNA gene amplicon sequencing [8–12], very limited evidence is available for the metabolic activities and microbial interactions therein. Although anammox and n-DAMO species are with determined functions, it is unclear whether they indeed take the active role in methane and nitrogen transformation. Additionally, the communities are always accompanied by flanking partners with functions to be explored, for instance, the members of the phyla Verrucomicrobiota and Bacteroidota that were found in nearly all the consortia of anammox and n-DAMO microorganisms. What are their roles and how do they survive in these systems remain to be answered. Answering these questions will be conducive to draw a comprehensive picture of this innovative and industrially important nitrogen removal process, which obviously possesses more complicated community structures and nuanced metabolic interactions in comparison to the well-studied anammox systems [13, 14].

In this study, we combined metagenomics and metatranscriptomics to reveal the microbial ecology of two biofilm systems, which coupled anammox and n-DAMO species for efficient nitrogen removal in low-strength domestic sewage and high-strength sidestream wastewater, respectively. The results of 16S rRNA gene amplicon, metagenomic and metatranscriptomic sequencing identified different community structures and metabolic interactions in the two examined systems, which was likely due to different nitrogen loadings applied. The reconstructed metabolic networks suggested the cross-feeding of various organic compounds between populations, likely supporting the survival of non-methanotrophic heterotrophs in these methane-fed systems. More interestingly, the study also offered the first transcriptional insight into a previously neglected possibility of “*Ca*. Methylomirabilis” bacteria in performing nitrate-dependent anaerobic methane oxidation and achieving complete denitrification by its own.

## Materials and methods

### Experimental setup and sampling strategy

Two identical biofilm systems (High_BS and Low_BS) inoculated with the same enriched suspended culture described by Haroon et al. [2] have been operated for more than 5 years. These two systems were fed with the synthetic medium with different concentrations of nitrite and ammonium to mimic high-strength sidestream wastewater and low-strength domestic sewage, respectively (Fig. S1, Tables S1 and S2) [5, 6]. The total nitrogen surface loading rate was ~2.0 g and ~0.2 g N/m^2^/d in High_BS and Low_BS, respectively, which was the only difference between the two systems across the operation (Supplementary Text S1). Each system has a total volume of ~2.3 L, with 12 bunches of hollow fibre membrane (Teijin, Japan) to supply methane and to support biofilm development. The systems were operated under anoxic conditions, with dissolved oxygen below the detection limit (0.6 μM). The pH in both systems was maintained at 6.8–7.2.

The community structure of both systems was monitored with periodic 16S rRNA gene amplicon sequencing, which showed relatively stable microbial communities (Fig. S2). Details for the 16S rRNA gene amplicon sequencing and analysis are described in Supplementary Text S2. Since no suspended biomass was observed in the bulk liquid, two biofilm samples (10 mL) were collected from each reactor for DNA extraction with a time interval of one year (Day 730 and 1095) for metagenomic sequencing. Biofilm samples collected on Day 1095 from each reactor were also used for metatranscriptomic sequencing (Table S3).

### Metagenomic sequencing and assembly

Total DNA was extracted as per the manufacturer’s protocol using FastDNA SPIN Kit for Soil (MP Biomedicals, Santa Ana, CA, USA). Libraries for metagenomic sequencing were prepared according to the manufacturer’s protocol using Nextera XT Library Preparation Kit (Illumina #FC-131-1096). Library preparation and beads clean-up were run on the Mantis Liquid Handler (Formulatrix) and Epimotion (Eppendorf #5075000301) automated platform. Libraries were quantified and quality control was performed using the QubitTM dsDNA HS Assay Kit (Invitrogen) and Agilent D5000 HS tapes (#5067-5592) on the TapeStation 4200 (Agilent #G2991AA). The sequencing pool was created with equimolar amounts of 1 nM per library and quantified in triplicates using the QubitTM dsDNA HS Assay Kit (Invitrogen). The libraries were sequenced on the NextSeq500 (Illumina, USA) using NextSeq 500/550 High Output v2 2x150bp paired-end chemistry at the Australian Centre for Ecogenomics (ACE). Generated reads were quality-checked using FastQC-v0.11.7 (Simon Andrews, http://www.bioinformatics.babraham.ac.uk/projects/fastqc/). Duplicates were removed using FastUniq-1.1 [15], and the remaining reads were trimmed using Trimmomatic 0.36 [16]. Reads shorter than 100 bp were discarded.

### Recovery of genomes, annotation and phylogenetic analysis

Co-assembly was performed using MEGAHIT-v1.1.3 [17]. Assembled contigs were further scaffolded using SSPACE-STANDARD 3.0 [18] with insert size estimated by mapping trimmed reads to assemblies with Bowtie 2.3.4.3 [19]. Gaps were further resolved using Abyss-sealer from ABySS 2.2.4 [20]. The obtained assemblies for High_BS and Low_BS consisted of 194,882 and 234,960 scaffolds (≥500 bp), with N50 of 4449 and 3584, respectively. Differential coverage binning was undertaken by mapping reads to the assemblies using Bowtie 2.3.4.3 [19] and SAMtools 1.10 [21]. Alignments with aligned length ≤ 90% of read length or with an identity ≤ 0.97 were removed. Then metagenome-assembled-genomes (MAGs) were recovered using MetaBAT v2.12.1 [22] with default settings. The generated MAGs were dereplicated using the ‘dereplicate’ workflow from dRep v2.4.0 [23] with primary and secondary average nucleotide identity (ANI) thresholds of 0.95 and 0.99, respectively. Taxonomic affiliations of MAGs were determined using workflow “classify_wf” from GTDB-Tk v1.0.2 [24]. The relative abundance of each MAG was calculated based on the ratio of mapped reads of each MAG to the total trimmed reads of each sample. The workflow “lineage_wf” from CheckM v1.1.0 [25] was used to determine the completeness and contamination of each MAG. Scaffolds within genomes with divergent GC ratio, coverage or tetranucleotide frequencies were removed using RefineM v0.1.1 (“--gc_perc 95 --td_perc 95 -e 0.03 -a 0.9”; Donovan Parks, https://github.com/dparks1134/RefineM).

For each MAG and unbinned scaffold, open reading frames (ORFs) calling and preliminary annotation were performed using Prokka 1.14.5 with domain information [26]. Additional annotation was conducted using different databases with details described in Supplementary Text S3.

To further investigate the phylogenetic placement of recovered MAGs (completeness > 75% and contamination < 10% by CheckM [25]), GTDB v89 was used to build the genome tree with 122 archaeal-specific (https://data.ace.uq.edu.au/public/gtdb/data/releases/release89/89.0/ar122_individual_genes_r89.tar.gz) and 120 bacterial-specific (https://data.ace.uq.edu.au/public/gtdb/data/releases/release89/89.0/bac120_individual_genes_r89.tar.gz) conserved marker genes. The evolutionary relationship for n-DAMO bacterial genomes recovered here was assessed with the construction of a genome-based phylogenetic tree for MAGs assigned to the NC10 phylum (see Supplementary Text S4 for details).

### Metatranscriptomic sequencing and analysis

Total RNA was extracted using the RNeasy Mini Kit (QIAGEN, Germany). DNase treatment was conducted using Turbo DNA-free kit (Ambion). Ribosomal RNA was depleted in these samples with the Illumina RiboZero Bacterial kit. Libraries were prepared with Illumina ScriptSeq v2. Quality control was performed for each library as aforementioned for metagenomic libraries. Qualified libraries were sequenced on Illumina NexSeq500 (Illumina, USA) with an average insert length of 300 bp at ACE.

Generated reads were quality-checked using FastQC-v0.11.7 and cut into 75 bp. Reads trimming was performed using Trimmomatic-0.36 [16]. Predicted rRNA reads were removed using SortMeRNA 4.2.0 [27] with a self-constructed database comprising 5SRNAdb [28], and SILVA v138 [29]. The remaining mRNA reads were mapped to dereplicated MAGs and unbinned scaffolds using Bowtie2. Alignments with aligned length <95% of read length or identity <97% were removed. Mapped reads of each coding sequence (CDS) were tabulated using RNAdir (Jie Li, https://github.com/jlli6t/RNAdir), which is a modified version of dirseq (Ben J. Woodcroft, https://github.com/wwood/dirseq). Expression levels for each CDS were calculated as Transcripts per million (TPM) [30]. The relative contribution of each species to the metatranscriptome was calculated as the sum of TPM for all CDS encoded by its representative MAG.

The relative expression of pathways was calculated as the average expression of each of the reactions of the pathway, where the expression of each enzymic reaction was firstly calculated as the average expression of genes encoding its subunits (e.g., expression of *pmoCAB* was calculated as the average TPM of subunit C, A and B). For reactions catalysed by multiple alternate enzymes, the expression was calculated as the sum-up of all these genes (e.g., nitrate reduction = *nar* + *nap* TPM).

### Statistical analysis

The 16S rRNA amplicon sequencing profiles were used for PCA analysis using the Python package scikit-learn. Visualisation of the data was performed using the matplotlib and seaborn Python packages. Alpha diversity of each sample was calculated according to Eq. (1) for both 16S rRNA gene sequencing data, metagenomic and metatranscriptomic data. Mann–Whitney U hypothesis testing was conducted, with null hypothesis H_0_: ${\upmu}_{High\_BS} = {\upmu}_{Low\_BS}$, to compare the median Shannon index values of High_BS and Low_BS using Python package SciPy.

1 $$Shannon\,Index\left( H \right) = - \mathop {\sum }\limits_{i = 1}^n p_i \ast \ln p_i.$$

where p is the proportion of individuals of one specific OTU/pathway divided by the total of individuals; n is the number of OTUs/pathways.

### Etymology

#### “*Candidatus* Methylomirabilis nitratireducens”

nitratireducens (ni.tra.ti.re.du’cens. N.L. masc. n. nitras (gen. nitratis), nitrate; L. pres. part. reducens, converting to a different state; N.L. part. adj. nitratireducens, reducing nitrate). This name implies an organism capable of consuming methane and reducing nitrate.

#### “*Candidatus* Methylomirabilis nitritireducens”

nitritireducens (ni.tri.ti.re.du’cens. N.L. masc. n. nitris (gen. nitritis), nitrite; L. pres. part. reducens, converting to a different state; N.L. part. adj. nitritireducens, reducing nitrite). This name implies an organism capable of consuming methane and reducing nitrite.

## Results and discussion

### Nitrogen removal performance and microbial activities

**Fig. 1:**
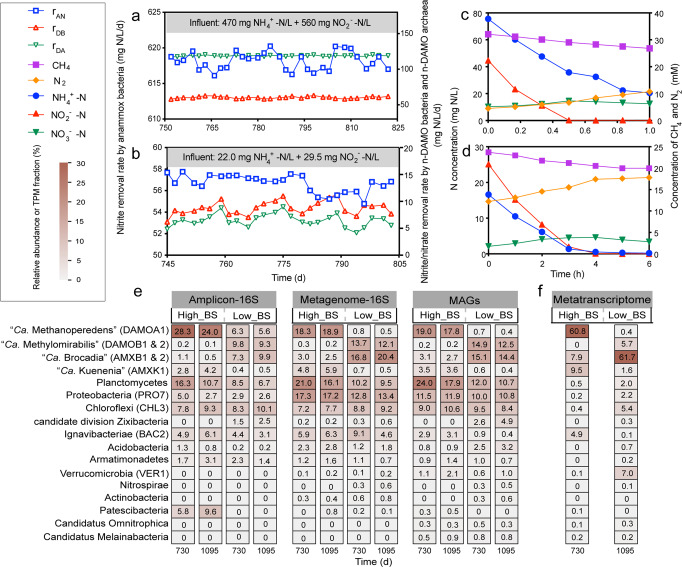
Nitrogen removal performance and microbial community profiles of High_BS (high loading) and Low_BS (low loading). Species-specific nitrogen conversion rates during the long-term operation of High_BS (**a**) and Low_BS (**b**): nitrite removal rate by anammox bacteria (r_AN_), nitrite removal rate by n-DAMO bacteria (r_DB_), and nitrate removal rate by n-DAMO archaea (r_DA_). Batch tests were performed on day 974 to validate the variations of nitrogenous substrates and methane of High_BS in 1 h (**c**) and of Low_BS in 6 h (**d**). **e** Microbial community structures of High_BS and Low_BS were revealed from 16S rRNA gene amplicon sequencing data, 16S rRNA genes from the metagenomic reads and recovered MAGs. **f** Relative expression of MAGs. DAMOA1 is the only MAG of n-DAMO archaea affiliated with “*Ca*. M. nitroreducens”, while DAMOB1 and DAMOB2 are the MAGs of n-DAMO bacteria belonging to “*Ca*. Methylomirabilis”. AMXK1, AMXB1 and AMXB2 are anammox MAGs. BAC2, VER1, CHL3 and PRO7 are MAGs with expression above 1% in either High_BS or Low_BS, belonging to Bacteroidota, Verrucomicrobiota, Chloroflexota and Proteobacteria, respectively. Numbers in the cell indicate the relative abundance or TPM fraction.

Fed with high-strength and low-strength wastewater, respectively, consistently low concentrations of ammonium, nitrite and nitrate were observed in the effluent of both the High_BS and Low_BS systems (Fig. S1a, b). In specific, the effluent nitrite was below 1 mg N/L in both systems, while the effluent ammonium and nitrate fluctuated but were consistently below 5 mg N/L. This good effluent quality suggested that both systems could effectively remove more than 95% of the total nitrogen in the feed, giving effluent total nitrogen concentration below 5 mg N/L and meeting the standard in most regions. Based on the applied hydraulic retention time (HRT) of 1 d for High_BS and 0.5 d for Low_BS, the volumetric nitrite removal rate by anammox bacteria (r_AN_) was determined to be 615–620 mg N/L/d and 55–60 mg N/L/d, respectively (Fig. 1a, b). Likewise, the nitrite removal rate by n-DAMO bacteria (r_DB_) and the nitrate removal rate by n-DAMO archaea (r_DA_) also stabilised during the long-term experiment, suggesting that both systems were operated at a steady state. Moreover, the cycling studies conducted on day 974 also showed that dinitrogen gas was produced in both systems along with the consumption of methane, nitrite, and ammonium, while nitrate accumulated initially but decreased when nitrite was fully consumed (Fig. 1c, d). Of note, the measured methane consumption rate was statistically close to that predicted by the reactions of anammox bacteria, n-DAMO bacteria and n-DAMO archaea (Text S1 and Table S1), which strongly suggested that these three microbial reactions were the key reactions in both systems.

### Influence of nitrogen loading on microbial community composition and activity

Periodical 16S rRNA gene amplicon profiles showed the dominance/existence of n-DAMO and anammox populations in both systems (Fig. 1e), while PCA analysis showed distinct communities in the High_BS and Low_BS systems (Fig. S2). Metagenome-based community profiles of biofilm samples collected on Days 730 and 1095 were consistent with amplicon sequencing-based profiles (Fig. 1e). In total, 56 non-redundant MAGs (≥75% completeness and ≤10% contamination) spanning 19 phyla were recovered (Tables S3 and S4, Figs. S3 and S4). These MAGs accounted for 72.8–77.6% of the total quality-trimmed reads of each sample (Table S5), indicating recovery of the majority of both communities.

Anammox and n-DAMO populations were prevalent in both systems, yet in different abundances (Fig. 1e). For instance, the AMXK1 affiliated with “*Ca*. Kuenenia” showed a slightly higher average relative abundance in High_BS (3.6%) than in Low_BS (0.5%), while the inverse was true for AMXB1 affiliated with “*Ca*. Brocadia” which dominated Low_BS (14.5%) but not High_BS (1.7%). Likewise, two genomes of n-DAMO bacteria (DAMOB1 and DAMOB2) affiliated with “*Ca*. Methylomirabilis” were identified at substantially lower abundances in High_BS (<0.2%) as opposed to Low_BS (12.4% and 1.3%). Notably, n-DAMO archaea “*Ca*. M. nitroreducens” (DAMOA1) known to couple anaerobic oxidation of methane (AOM) to nitrate reduction [2], existed in both systems and dominated the High_BS community with an average relative abundance of 18.4%. As both systems were fed with only ammonium and nitrite, this indicated an interesting reciprocal feeding between n-DAMO archaea and anammox bacteria. Anammox bacteria supply n-DAMO archaea with nitrate (NH_4_^+^ + 1.32NO_2_^-^ → 1.02N_2_ + 0.26NO_3_^-^ + 2.03H_2_O) and receive nitrite in return (CH_4_ + 4NO_3_^-^ → CO_2_ + 4NO_2_^-^ + 2H_2_O).

To further investigate the active populations in communities, metatranscriptomic sequencing was performed on biofilms collected from Day 1095 for both communities (Table S3). Expression of MAGs was profiled as TPM fractions (Fig. 1f). Active MAGs were defined as those having TPM fraction >1%, which collectively accounted for >82% of the total TPM in both communities and were therefore responsible for the bulk of the observed carbon and nitrogen transformations of both systems (Table S6).

With the same feeding composition but different loadings, substantial differences in the expression profiles were also observed between the two systems. For example, DAMOA1 was the most active species contributing 60.4% of the total expression in High_BS, followed by anammox bacteria AMXK1 (9.7%), AMXB1 (5.0%), and AMXB2 (3.0%). Conversely, AMXB1 dominated the expression profile of Low_BS (61.7%), while DAMOB1 (3.3%) and DAMOB2 (2.4%) were the most active methanotrophs. In addition, BAC2 was the only active side population in High_BS with a TPM of 5.1%, while VER1, CHL3 and PRO7 were active in Low_BS with TPM values of 7.1%, 4.1% and 1.4%, respectively.

Nitrogen loading, as the only difference between the two systems, shaped the phylogenetic and functional diversities of microbial communities. Alpha diversity analysis based on amplicon profiles revealed a significantly lower Shannon diversity in the high-loading High_BS system (2.76 ± 0.05) compared to the low-loading Low_BS system (3.25 ± 0.47; *p* < 0.05). The same pattern of Shannon diversity was confirmed based on the metagenomic reads (High_BS, 9.64–9.87; Low_BS, 10.12–10.44). Based on the metatranscriptomic data, a higher functional diversity was also observed for the active community of the Low_BS compared to the High_BS (Shannon diversity of 4.03 and 3.72, respectively).

### Diversity and genomic features of bacteria belonging to “*Ca*. Methylomirabilis”

**Fig. 2:**
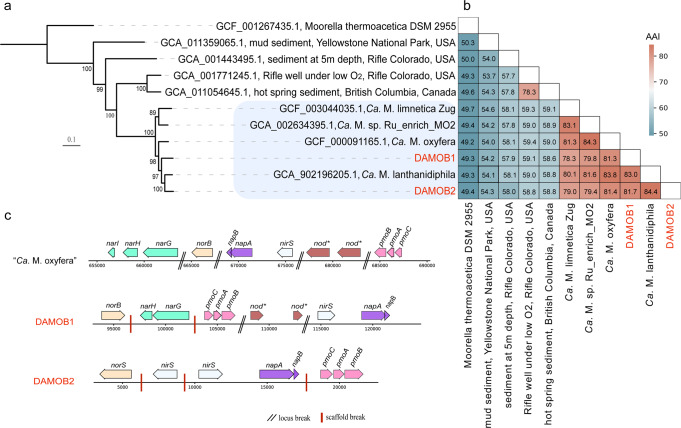
Phylogenetic and genomic features of n-DAMO bacteria. **a** Phylogenetic placement of the two n-DAMO bacteria MAGs recovered in this study (DAMOB1 and DAMOB2) using 120 bacterial-specific marker genes with publicly available non-redundant candidate division Methylomirabilota genomes (see method for details). The tree was inferred using the maximum-likelihood method, and bootstrap values were calculated using nonparametric bootstrapping with 100 replicates. The scale bar represents amino acid substitutions per site. The shaded area is clade a of Methylomirabilota phylum. **b** Average amino acid identity (AAI) as indicated by the values in cells. **c** Schematic representation showing the arrangement of genes for denitrification and methane oxidation in two recovered genomes of n-DAMO bacteria in the present study and “*Ca*. M. oxyfera” (GCF_000091165.1). Arrows represent genes and indicate the transcriptional direction.

Two recovered high-quality genomes (DAMOB1 and DAMOB2) are classified within the phylum Methylomirabilota. Phylogenetical placement suggested that DAMOB1 is phylogenetically close to “*Ca*. M. oxyfera” [4], while DAMOB2 is close to “*Ca*. M. lanthanidiphila” [31] (Fig. 2a). Comparative genome analyses revealed an average amino acid (AAI) < 85% between the two recovered MAGs and genomes in the public database (Fig. 2b), indicating that each of the two MAGs likely represents novel species [32].

Similar to the widely known “*Ca*. M. oxyfera” [4], both DAMOB1 and DAMOB2 expressed *pmo* genes for methane oxidation and relevant genes for nitrite reduction, but did not encode the traditional nitrous oxide reductase for N_2_ production (Fig. 2c). Interestingly, DAMOB1 also expressed nitrate reductases *narGH* and *napAB* above the median expression value, suggesting its potential to perform nitrate reduction. While nitrate reductase genes were also detected in other published “*Ca*. Methylomirabilis” genomes (e.g., “*Ca*. M. oxyfera” [4], “*Ca*. M. lanthanidiphila” [31], and “*Ca*. M. limnetica” [33]), the current study provides the first expressional evidence of nitrate reductase by a species within the phylum Methylomirabilota. This result supports the potential capability of Methylomirabilota members to perform nitrate-dependent AOM, which requires further investigation to confirm. Analogous to “*Ca*. M. oxyfera” and “*Ca*. M. lanthanidiphila”, DAMOB1 encoded two putative *nod* genes potentially for oxygen generation via nitric oxide dismutation [4, 31]. These *nod* genes were among the highest expressed genes, indicating their active role (Fig. 2c). However, no *nod* gene was annotated in DAMOB2, which is unlikely due to assembly or binning, considering the high completeness of the MAG (95.4%). Without the putative *nod* gene for NO dismutation, the source of oxygen for DAMOB2 to activate methane oxidation is a mystery. Given the fact that the function of the so-called NOD enzyme remains to be experimentally proven [34], there might be other unknown genes catalysing the dismutation of NO and generation of oxygen.

Drawing upon the inferred functions of DAMOB1 and DAMOB2, we suggest designating these two new species as “*Ca*. Methylomirabilis nitratireducens” and “*Ca*. Methylomirabilis nitritireducens”, representing their capability in performing nitrate-dependent and nitrite-dependent anaerobic methane oxidation, respectively. Since the initial discovery of relevant microorganisms in 2006 [3], the methane-dependent complete denitrification is believed to be carried out by a consortium of microorganisms, during which archaeon from “*Ca*. Methanoperedens” is responsible for the nitrate reduction to nitrite, while bacterium belonging to “*Ca*. Methylomirabilis” further reduces nitrite to dinitrogen gas. The present study challenges this understanding by identifying “*Ca*. Methylomirabilis nitratireducens” and highlighting its highly expressed complete denitrification pathway from nitrate to dinitrogen gas within a single microorganism, rather than across multiple organisms.

### Varying nitrogen and methane metabolic pathways under high and low nitrogen loadings

**Fig. 3:**
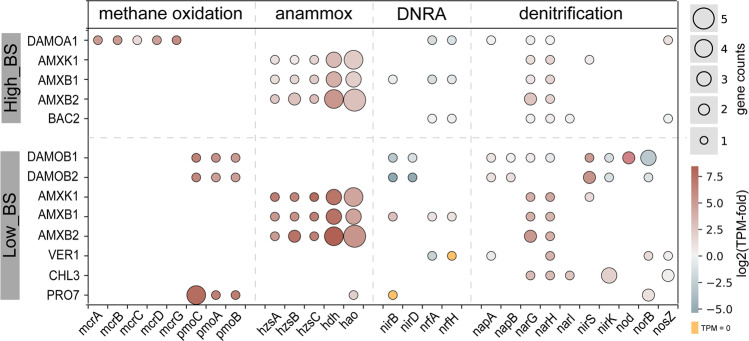
Relative expression of genes involved in major methane and nitrogen metabolic pathways encoded by each MAG. Colour intensity represents gene expression. Gene expression was relativized by median TPM value across all CDS within a given genome (see details in Methods). A value of one means two times of median expression among all CDS in a given genome. Orange cell indicates the expression of the encoded gene is undetectable. Bubble diameter represents the gene count. Only the MAGs with expression >1% were presented. The TPM values of encoded genes by each MAG were listed in Supplementary Dataset 2.

The impact of nitrogen loading on metabolic pathways for nitrogen and methane conversions was further investigated (Fig. 3 and Supplementary Dataset 1). DAMOA1 expressed methyl coenzyme M reductase (*mcrABCDG*), enabling the reverse methanogenesis pathway [2], while DAMOB1 and DAMOB2 expressed particulate methane monooxygenase (*pmoCAB*), suggesting the intra-aerobic methane oxidation pathway [4]. Unexpectedly, PRO7 affiliated with *Methylocystis* (typically known as an aerobic methanotroph) also expressed *pmo* genes in Low_BS despite no oxygen supplied or detected in the system. One of the possibilities for the survival of PRO7 is scavenging trace oxygen that is below the detection limit (0.6 μM), as aerobic methane oxidation can occur at oxygen even below 0.5 μM [35].

The genes relevant to nitrogen transformations were categorised into three groups: anammox, DNRA, and denitrification. Consistent with previous studies [2, 36], DAMOA1 highly expressed the *narGH* for nitrate reduction to nitrite and dissimilatory periplasmic cytochrome *c* nitrite reductase genes (*nrfHA*) for DNRA, while DAMOB1 and DAMOB2 mainly expressed nitrite reductase (*nirS*) gene for nitrite reduction. Meanwhile, the central genes involved in anammox metabolism were among the highest expressed genes in the three anammox genomes (AMXK1, AMXB1 and AMXB2), such as the hydrazine synthases (*hzsABC*), hydrazine dehydrogenase (*hdh*) and hydroxylamine oxidoreductase (*hao*). Similar to the reported “*Ca*. K. stuttgartiensis” [37], “*Ca*. B. sinica” [38] and “*Ca*. B. sapporoensis” [39], *narGH* genes slightly expressed in the three anammox MAGs, which might contribute to nitrate reduction to nitrite. Moreover, neither *nirS* nor copper-containing nitrite reductase (*nirK*) was encoded in the AMXB1 or AMXB2 MAGs. This may support the proposed hydroxylamine-dependent anammox mechanism in “*Ca*. B. sinica”, in which nitrite was first reduced to hydroxylamine rather than nitric oxide [40].

Furthermore, side populations appeared to perform different functions in the two systems. Specifically, BAC2 (belonging to Bacteroidota), the only active side population in High_BS treating high-strength wastewater, expressed *nar* and *nrf* genes, indicating their potential for DNRA. In Low_BS fed with low-strength wastewater, VER1 (affiliated with Verrucomicrobiota) also potentially performed DNRA, while CHL3 (affiliated with Chloroflexota) expressed genes for the reduction of nitrate to nitric oxide, mediated by *narGHI* and *nirK*. Interestingly, no recovered non-methanotrophic genome was found to encode the complete denitrification pathway from nitrate to dinitrogen gas (Fig. 3). As also observed in anammox engineered systems [13, 14] and natural environments such as aquifer [41] and estuary sediment [42], the interspecific cross-feeding of substrates may be critical for these microbial communities. However, where these non-methanotrophic heterotrophs acquire electrons and carbons remains to be further investigated.

### Potential cross-feeding of amino acids and methane-derived intermediates

**Fig. 4:**
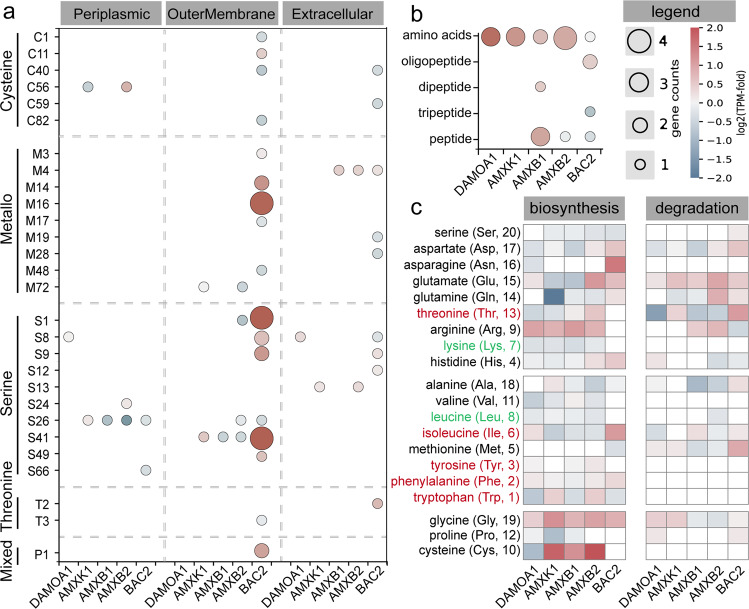
Expression of predicted peptides, transporters and amino acids biosynthetic and degradation pathways encoded by the active populations in high-nitrogen loading system High_BS. **a** Gene count (bubble diameter) and expression (colour intensity, compared to median gene expression of genome) of peptidases possibly involved in EPS matrix degradation across active MAGs. The subcellular location of peptidases was predicted using the subcellular localisation predictor (CELLO 2.5) [55]. **b** Gene count (bubble diameter) and expression (colour intensity, compared to median gene expression of MAGs) of amino acid, peptide and protein transporters across active genomes. **c** Presence and expression of amino acid biosynthesis and degradation pathways across active MAGs. Bracketed numbers rank the metabolic cost of amino acid biosynthesis based on [45], with 1 being the most costly. Top, middle and bottom panels include amino acids that are hydrophilic, hydrophobic and with special structured side chains, respectively. Amino acid types of glucogenic, ketogenic or both are highlighted in black, green and red, respectively. A detailed summary can be found in Supplementary Dataset 2.

Extracellular polymeric substances (EPS) which are widely present in biofilms are a potential source of carbon for heterotrophic side populations (e.g., BAC2 in High_BS and VER1 and CHL3 in Low_BS). In both the high- and low-nitrogen loading systems, a variety of peptidases were actively expressed by heterotrophic populations (Figs. 4a and S5a). Specifically, BAC2 in the High_BS community highly expressed extracellular serine-like peptidases, metallopeptidases, and cysteine-like peptidases (Fig. 4a), while VER1 and CHL3 highly expressed these categories of periplasmic peptidases in the Low_BS community (Fig. S5a). In line with the observed ability to hydrolyse proteins into peptides or amino acids, side populations expressed corresponding genes to transport these small organic molecules across the cell membrane (Figs. 4b and S5b) and to utilise them as carbon and energy sources. Interestingly, the glucogenic amino acids were, in general, more likely to be catabolized in comparison to ketogenic amino acids (Figs. 4c and S5c), suggesting that amino acids were preferentially used through the glycolysis pathway instead of the TCA cycle [43, 44]. Furthermore, the degradation of amino acids that require high biosynthetic cost (e.g., tryptophan, phenylalanine and tyrosine) was limited [45], whereas their biosynthesis was comparable (Fig. 4c). Of note, tryptophan, phenylalanine and tyrosine carry a benzene ring, while the rest amino acids only contain six or fewer carbons per molecule. This suggests that amino acids with fewer carbon atoms tend to be catabolized directly, while others are possibly used for protein synthesis [13].

Additionally, side populations also lacked the biosynthesis pathway for a variety of necessary amino acids (Figs. 4c and S5c), e.g., threonine, arginine, lysine and proline, indicating the cross-feeding from other members who carry the corresponding biosynthesis pathway, such as n-DAMO and anammox microorganisms. These observations together suggest that proteinaceous substrates in EPS and amino acids might serve as carbon sources for the heterotrophic side populations. Indeed, the genetic potential for proteinaceous substrate hydrolysis, transport and breakdown of peptides and amino acids was detected in nearly all abundant populations of the two systems, indicating the exchange of these organic intermediates might be more general than expected.

Methane-derived organic compounds might serve as alternative intermediates exchanged among the active species. DAMOA1 was the main methanotroph in High_BS, which expressed the pathway for reversible conversion of poly-β-hydroxybutyrate (P3HB, one type of polyhydroxyalkanoate) and acetate via acetyl-CoA. This genomic speculation is supported by our previous experiment, which has shown the correlation between acetate production and polyhydroxyalkanoate degradation in an enriched “*Ca*. M. nitroreducens” culture when nitrate/nitrite was depleted [46]. To be noted, the *in situ* nitrate/nitrite concentration was consistently below 5 mg N/L in present systems (Table S2), forming a substrate-limited condition that may favour the release of acetate from polyhydroxyalkanoate [46]. In the Low_BS community, the methane oxidation mainly performed by bacterial methanotrophs reportedly leads to the generation of various organic intermediates under oxygen limitation, such as methanol, lactate, formate, and acetate [47–50]. Given the only source of oxygen in the Low_BS would be from the nitric oxide dismutation catalysed by NOD of n-DAMO bacteria [4], the oxygen-limited condition may favour the release of aforementioned intermediates by the prevalent bacterial methanotrophs. These simple methane-derived organic molecules could be easily utilised by most heterotrophs as energy and carbon source. This is also supported by the expression of a number of related genes and pathways by side populations, including acetyl-CoA synthetase (*acs*), phosphate acetyltransferase (*pta*)-acetate kinase (*ack*) pathway, lactate dehydrogenase (*ldh*), and methanol-5-hydroxybenzimidazolylcobamide Co-methyltransferase (*mtaB*).

### Microbial competition and synergy

**Fig. 5:**
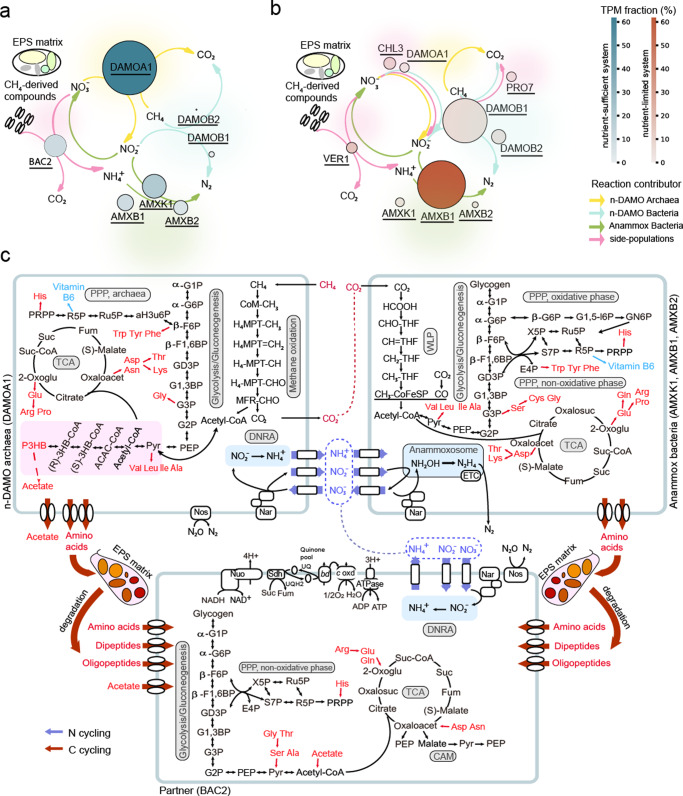
Schematic of metabolic networks. **a** Schematic of microbiota metabolic network under high-loading and **b** low-loading conditions: bubble size indicates the relative abundance, while the colour intensity indicates the expression fraction. **c** Integrating metabolic network related to nitrogen and methane conversions in high-loading system High_BS: blue arrows indicate nitrogen cycling, while red arrows indicate carbon cycling.

The conversions of nitrogen and methane are driven by microbial competition and synergy (Fig. 5a, b). Kinetics are considered the key to microbial competition. Although the methane affinity constant of n-DAMO archaea is at least one order of magnitude higher than n-DAMO bacteria of 2.6 ± 0.7 μM [51, 52], the competition for methane is not considered critical as it was continuously and efficiently supplied to both systems. In the competition for nitrite, anammox bacteria were observed to outcompete n-DAMO bacteria in a suspended culture [2], due to their higher affinity to nitrite [52, 53], which, however, co-existed in High_BS and Low_BS communities in the present study. This may be explained by the specific counter-diffusion of substrates in biofilm matrix. With methane supplied from the inner layer of biofilm (i.e., from membrane-biofilm interface) and nitrogen compounds (ammonium and nitrite) offered from the outer layer of biofilm (i.e., from biofilm-water interface), a middle region was created where ammonium was depleted while methane and nitrite were available. Thus, n-DAMO bacteria can inhabit this particular niche rather than being completely washed out from the biofilm, as validated by previous mathematical modelling results [7]. This is also consistent with our recent findings of microbial stratification in biofilm, showing that n-DAMO archaea, n-DAMO bacteria, and anammox bacteria dominated the inner layer, middle layer, and outer layer of biofilms, respectively [54].

The reconstructed metabolic networks suggest the critical role of microbial synergy, during which various substrates are exchanged interspecifically to fulfil their respective growing requirement (Figs. 5c and S6). For example, nitrogen compounds are interspecifically exchanged in both systems. Anammox bacteria were the main consumer of ammonium and nitrite in the feeding in both communities, with nitrate as a product. The generated nitrate could be utilised by other members, such as n-DAMO archaea and partner BAC2 in High_BS (Fig. 5c), and n-DAMO bacteria and partner CHL3 in Low_BS (Fig. S6). In addition, DNRA was carried out by members of both communities, including BAC2 in the high-loading system and VER1 in the low-loading system, replenishing ammonium which was then reciprocally cross-fed to anammox bacteria.

Apart from nitrogen compounds, various carbon compounds were also interspecifically exchanged. Methane, the only exogenous organic carbon, was oxidised by multiple methanotrophs, such as n-DAMO archaea in High_BS, and n-DAMO bacteria and partner PRO7 in Low_BS. While the end product carbon dioxide could be utilised by anammox bacteria for autotrophic growth through the highly expressed Wood–Ljungdahl pathway, other organic intermediates (e.g., acetate, lactate, and methanol) could potentially support heterotrophic members, so well as proteinaceous substrates in EPS and amino acids. Two sources of amino acids were proposed in the network, which could be from the EPS breakdown and cell secretion by other dominating members (e.g., anammox bacteria). Moreover, the observed differentiation in catabolising glucogenic or ketogenic amino acids, and the amino acids with different molecule sizes, suggests that the exchange of amino acids is aptitudinal [43, 44].

### Summary

In recent years, the use of methane as the sole carbon source in the nitrogen removal process has shown great promise and industrial importance. In this study, we focused on the microbial ecology of biofilm communities involved in methane and nitrogen transformations. Our investigation provides the first genomic and transcriptomic evidence of the metabolic activities and interactions between anammox, n-DAMO, and other dominant side populations within these biofilm communities. The results substantiate the critical role of nitrogen loading in shaping the community structure, and the diversity of taxonomy and functionality. The cross-feeding of various nitrogen and carbon compounds among members of the community was found to be more common than expected. This may represent a potential phenomenon in the organic carbon-depleted and methane-bearing ecosystems (e.g., freshwater sediments), where active species supply carbon such as amino acids and methane-derived organic compounds to co-abundant heterotrophic populations. However, the intermediates were likely produced and consumed simultaneously, which makes their measurement difficult. Material-based evidence is expected to confirm the cross-feeding of organic intermediates in future studies.

Moreover, two novel “*Ca*. Methylomirabilis” genomes were recovered, which we designate as “*Ca*. M nitratireducens” and “*Ca*. M nitritireducens”, representing their inferred capability in performing nitrate-dependent and nitrite-dependent anaerobic methane oxidation, respectively. Of note, the finding of the highly expressed complete denitrification pathway from nitrate to dinitrogen gas by “*Ca*. Methylomirabilis nitratireducens” challenges the previous understanding that the methane-dependent denitrification is carried out by the consortium of bacteria and archaea. This is the first-ever transcriptomic evidence suggesting the potential of “*Ca*. Methylomirabilis” to perform nitrate reduction, which were previously believed to only conduct nitrite-dependent AOM.

## Supplementary information

Supplementary Information

Supplementary Dataset 1

Supplementary Dataset 2

Supplementary Dataset 3

## Data Availability

All sequencing data have been deposited in the NCBI SRA database under BioProject PRJNA796803. Amplicon samples are under Biosample accessions SAMN24966701-SAMN24966704. Metagenome samples are under Biosample accessions SAMN24919182-SAMN24919185. Metatranscriptome samples are under Biosample accessions SAMN24966675 and SAMN24966676.
