# Supplementary material for: Phylogenetic and metabolic diversity of microbial communities performing anaerobic ammonium and methane oxidations under different nitrogen loadings
Source: ISME Commun. 2023 Apr 25;3:39. doi: 10.1038/s43705-023-00246-4 (PMC10130057; doi:10.1038/s43705-023-00246-4)
Supplement: Supplementary file 1 — Supplementary Information [file 43705_2023_246_MOESM1_ESM.docx]

**Supplementary Information**

**Phylogenetic and metabolic diversity of microbial communities performing anaerobic ammonium and methane oxidations under different nitrogen loadings**

Jie Li^1^, Tao Liu^1🖂^, Simon J. McIlroy^2^, Gene W. Tyson^2^, Jianhua Guo^1🖂^

^1^Australian Centre for Water and Environmental Biotechnology, The University of Queensland, St Lucia, Queensland, Australia

^2^Centre for Microbiome Research, School of Biomedical Sciences, Queensland University of Technology, Translational Research Institute, Woolloongabba, Queensland, Australia

^🖂^Corresponding author email: [jianhua.guo@uq.edu.au](mailto:jianhua.guo@uq.edu.au); uqtliu8@uq.edu.au

**Text 1. Mass balance**

The consumption rate of substrate can be calculated as follows:

*r*_A_ = (*c*_A-eff_ - *c*_A-inf_) / HRT (1)

where *r*_A_ is the consumption rate of substrate A (mg N/L/d); *c*_A-eff_ and *c*_A-inf_ are the concentrations of substrate A in the effluent and influent, respectively (mg N/L); HRT is the hydraulic retention time (d). For example, the ammonium consumption rate (*r*_NH4+_) was negative because the ammonium concentration in effluent was always lower than that in influent.

Based on Reactions 1-3 above, the ammonium, nitrite and nitrate consumption rates (*r*_NH4+_, *r*_NO2-_ and *r*_NO3-_) can be written as follows:^1, 2^

*r*_NH4+_ = -1/1.32 * *r*_AN_ (2)

*r*_NO2-_ = - *r*_AN_ - *r*_DB_ + *r*_DA_ (3)

*r*_NO3-_ = - *r*_DA_ + 0.26/1.32 * *r*_AN_ (4)

where *r*_AN_ and *r*_DB_ (mg N/L/d) are the nitrite conversion rates by anammox bacteria and n-DAMO bacteria, respectively; *r*_DA_ (mg N/L/d) is the nitrate conversion rate by n-DAMO archaea. The microbial nitrogen conversion rate is absolute value.

Therefore, *r*_AN_, *r*_DB_ and *r*_DA_ can be calculated from the measured *r*_NH4+_, *r*_NO2-_ and *r*_NO3-_ as follows:

*r*_AN_ = -1.32 * *r*_NH4+_ (5)

*r*_DB_ = 1.06 * *r*_NH4+_ - *r*_NO2-_ - *r*_NO3-_ (6)

*r*_DA_ = -0.26 * *r*_NH4+_ - *r*_NO3-_ (7)

Based on the reaction stoichiometry, reducing 1 mg NO_2_^-^-N to N_2_ by n-DAMO bacteria requires 0.43 mg CH_4_, while reducing 1 mg NO_3_^-^-N to NO_2_^-^-N by n-DAMO archaea requires 0.29 mg CH_4_. Therefore, the predicted total methane consumption rate (*r*_CH4-predicted_, mg CH_4_/L/d) is calculated as:

*r*_DB-CH4_ = 0.43*r*_DB_  (8)

*r*_DA-CH4_ = 0.29*r*_DA_ (9)

**Text 2. 16S rRNA gene amplicon sequencing and analysis**

Primers 926F (5ʼ-AAACTYAAAKGAATTGACGG-3ʼ) and 1392R (5ʼ-ACGGGCGGTGTGTRC-3ʼ) were used for 16S rRNA gene amplification. PCR products were sequenced at the ACE. Raw sequencing reads were trimmed using Trimmomatic 0.36 ^1^. Trimmed reads were merged via overlap between pair-end reads using FLASH-1.2.11 ^2^ with parameters “-m 15 -x 0.1 -M 300”. Singletons were removed. Afterwards, left tags were clustered into OTUs using UPARSE-OTU algorithm ^3^ that integrated in command ‘cluster_otus’ of usearch10.0.240 ^4^ at 97% identity level. OTUs were classified using qiime 1.9.1 ^5^ with command “assign_taxonomy.py” with parameter “-m rdp” ^6^. During the classification process, SILVA ^7^ version 132 was used as reference.

**Text 3. Annotation and metabolic reconstruction of recovered genomes**

For each MAG, open reading frames (ORFs) calling and preliminary annotation were performed using Prokka 1.14.5 with domain information ^8^. Additional annotation was conducted using KEGG ^9^ (accessed 202003), eggNOG v5.0 ^10^ and Swiss-Prot (accessed 202003) ^11^. KofamKOALA 1.2.0 ^12^ was used to search KEGG Orthologs. Best hit of each gene with e < 1×e^-10^ and maximal F-score was chosen. Database eggNOG was searched using emapper 2.0.1-4-g2466c1b ^13^ in diamond mode with domain information. Hits with e > 1× e^-10^, or query coverage < 80%, or subject coverage < 50% were removed. DIAMOND 0.9.30 ^14^ was used to search against Swiss-Prot (accessed 202003). Best hit of each gene with e < 1× e^-10^, both query coverage and subject coverage > 50% were chosen. Finally, appropriate hits were mapped to the KO database. Transporters of interest were further annotated against TCDB (accessed 202010) ^15^ using diamond. Best hit with e < 1× e^-10^, both query coverage and subject coverage > 50% were chosen. Amino acids metabolism genes were imported into Pathway Tools and searched against MetaCyc ^16^ for amino acids biosynthesis and degradation construction. For the unbinned scaffolds, ORFs calling and preliminary annotation were performed using Prokka in metagenome mode. KEGG Orthologs searching was undertaken using KofamKOALA in the same process as aforementioned annotation of MAGs.

**Text 4. Phylogenetic analysis of recovered genomes**

To reveal the phylogenetic placement of recovered MAGs (completeness > 80% and contamination < 10% by checkM), GTDB v89 was used to build the genome tree with 122 archaeal-specific and 120 bacterial-specific conserved marker genes. Genes were predicted using Prodigal 2.6 ^17^. The conserved marker genes were identified and aligned using HMMER 3.3 ^17^. Concatenated and trees were constructed using FastTree 2.1.10 ^18^ with WAG+GMMA models. Bootstrap values were determined using GenomeTreeTk v0.1.2 (https://github.com/dparks1134/GenomeTreeTk). Trees were visualized using iTOL 5.5.1 ^19^ and imported into Adobe Illustrator for further refinement.

Moreover, in order to reveal the phylogenetic placement of the new n-DAMO bacterial genomes recovered in the present study, a genome tree for genomes in NC10 phylum was constructed. All available genomes that affiliated with phylum candidate division NC10 were collected from NCBI database accessed in April 2020. Retrieved genomes with completeness > 80% and contamination < 10% were retained, and then dereplicated using dRep v2.4.0 ^20^. The resulted genomes together with two NC10 MAGs recovered from present study were used to construct this genome tree using 120 bacterial-specific conserved marker genes. Bootstrap values were determined using GenomeTreeTk. Tree was visualized using iTOL and imported into Adobe Illustrator for refinement


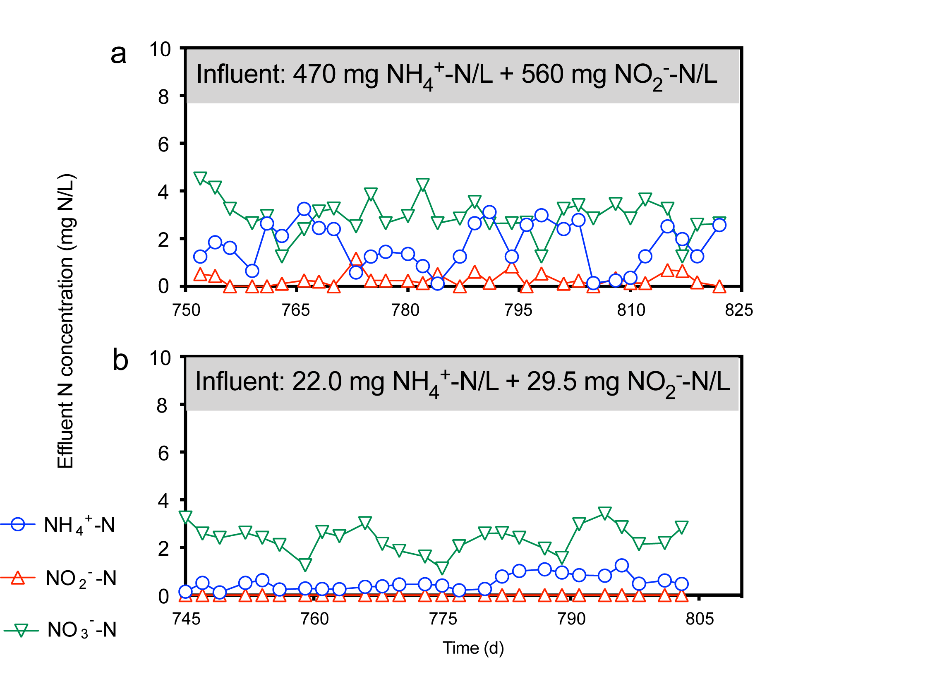


**Fig. S1** Long-term influent and effluent profiles of **a** high-loading and **b** low-loading reactors.


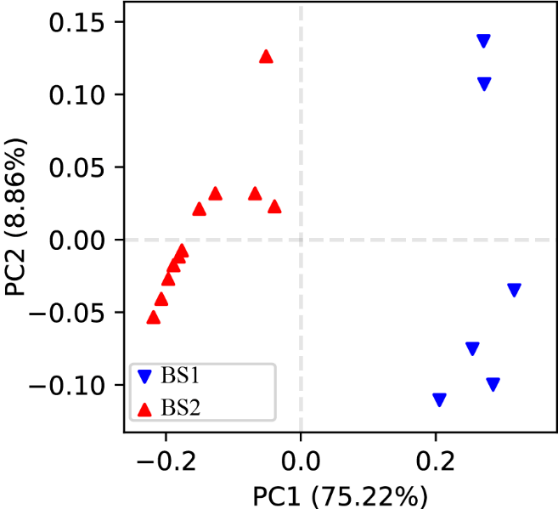


**Fig. S2 PCA analysis of microbial communities in high-loading High_BS and low-loading Low_BS.** A total of 6 and 11 16S rRNA gene amplicon sequencing-derived datasets were adopted from High_BS and Low_BS, respectively.

**Fig. S3 Phylogenetic placement of bacterial MAGs recovered from this study**. The bacterial genome tree was inferred using maximum-likelihood method with a concatenated set of 120 bacterial-specific marker genes. Bootstrap values were calculated using non-parametric bootstrapping with 100 replicates. Bacterial MAGs recovered from this study were highlighted in red. The scale bar represents amino acid substitutions per site.

**Fig. S4 Phylogenetic placement of archaeal MAGs recovered from this study**. The archaeal genome tree was inferred using maximum-likelihood method with a concatenated set of 122 archaeal-specific marker genes. Bootstrap values were calculated using non-parametric bootstrapping with 100 replicates. Archaeal MAGs recovered from this study was highlighted in red. The scale bar represents amino acid substitutions per site.

.


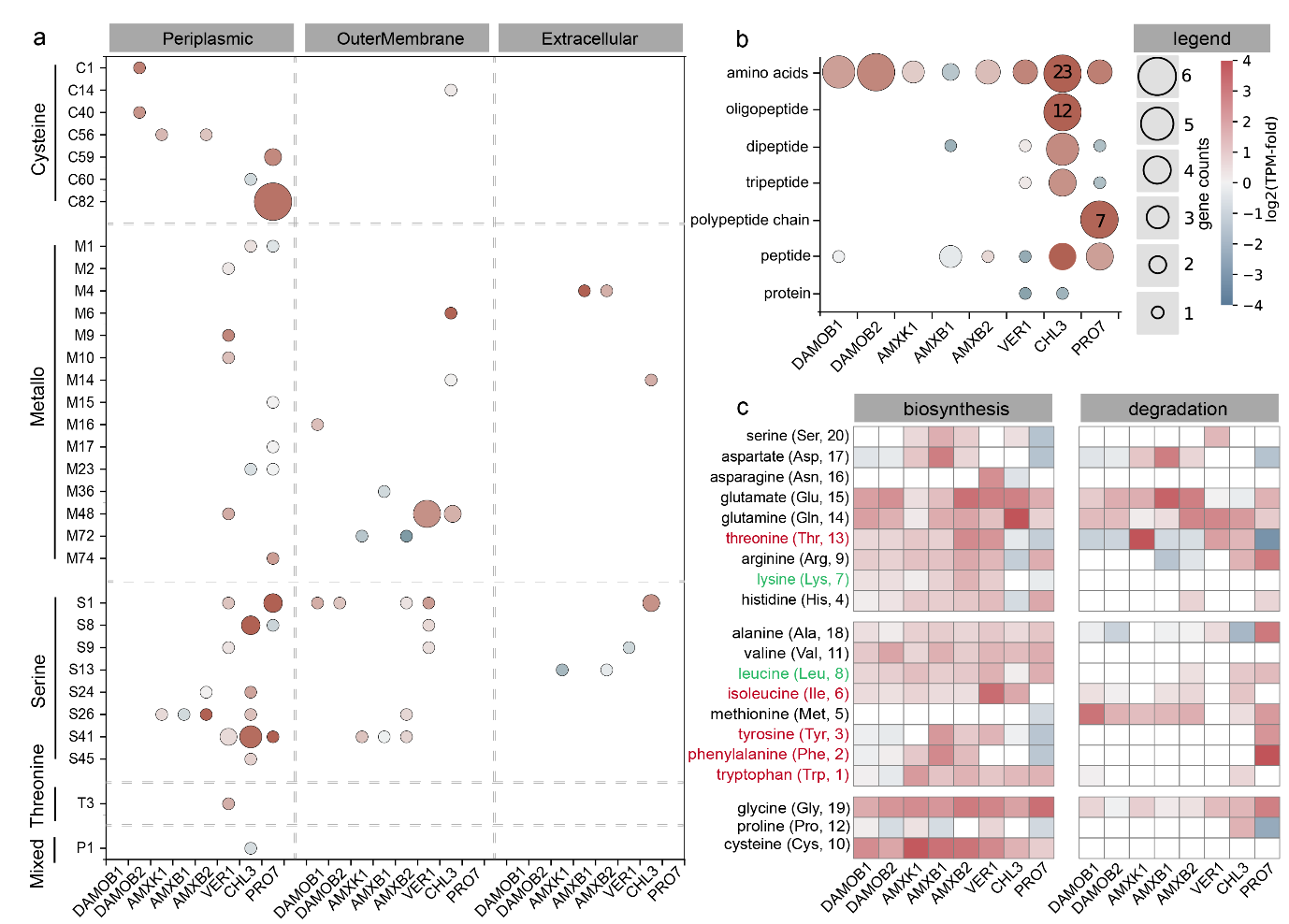


**Fig. S5 Predicted peptides, transporters and amino acids biosynthetic and degradation pathways encoded by the active populations in low-loading system Low_BS.** **a** Gene count (bubble diameter) and gene expression (color intensity, compared to median gene expression of genome) of peptidases possibly involved in EPS matrix degradation across active genomes. The subcellular location of peptidases was predicted using the subcellular localization predictor (CELLO 2.5) ^21^. **b** Gene count (bubble diameter) and gene expression (color intensity, compared to median gene expression of genome) of amino acid, peptide and protein transporters across active genomes. **c** Presence and expression of amino acid biosynthesis and degradation across active genomes. Bracketed numbers rank the metabolic cost of amino acid biosynthesis based on Akashi and Gojobori ^22^, with 1 being the most costly. Top, middle and bottom panels include amino acids that are hydrophilic, hydrophobic and with special structured side chains, respectively. Amino acid types of glucogenic, ketogenic or both are highlighted in black, green and red. A value of 1 in color key means 2 times of median expression in a given genome among all expressed genes. A detailed summary can be found in Supplementary Dataset 2.

**Fig. S6** Integrating metabolic network related to nitrogen and methane conversions in low-loading system Low_BS. Blue arrows indicate nitrogen cycling, while red arrows indicate carbon cycling.

Supplementary Tables

# Table S1. Measured ammonium, nitrite, nitrate and methane consumption rates in Batch Tests Set B.

| Unit  mg N L^-1^ h^-1^or mg CH_4_ L^-1^ h^-1^ | Measured | | | | Predicted |
| --- | --- | --- | --- | --- | --- |
|  | rNO_2_^-^ | rNO_3_^-^ | rNH_4_^+^ | rCH_4_ | rCH_4_-p |
| High_BS | -88.9 ± 5.8 | + 8.6 ± 2.6 | -59.7 ± 8.2 | -8.0 ± 1.5 | -9.4 ± 2.2 |
| Low_BS | -7.7 ± 3.1 | +0.8 ± 0.1 | -5.1 ± 1.2 | -0.8 ± 0.2 | -0.9 ± 0.3 |

**Table S2.** An overview of High_BS and Low_BS operated under high nitrogen loading and low nitrogen loading, respectively. The nitrogen compositions of influent and effluent were summarized, with sampling times for metagenomic and metatranscriptomic sequencing indicated.

| Biofilm System | Gas supplied | Influent | Effluent | DNA samples | RNA samples |
| --- | --- | --- | --- | --- | --- |
| High_BS | 95%CH_4_ + 5%CO_2_ | ~500 mg NH_4_^+^-N/L  ~500 mg NO_2_^-^-N/L | < 1 mg NH_4_^+^-N/L  < 1 mg NO_2_^-^-N/L  < 3 mg NO_3_^-^-N/L | Day 730  Day 1095 | Day 1095 |
| Low_BS | 95%CH_4_ + 5%CO_2_ | ~25 mg NH_4_^+^-N/L  ~25 mg NO_2_^-^-N/L | < 3 mg NH_4_^+^-N/L  < 1 mg NO_2_^-^-N/L  < 3 mg NO_3_^-^-N/L | Day 730  Day 1095 | Day 1095 |

**Table S3.** **Primary statistics of metagenomic and metatranscriptomic sequencing data**.

|  | **Sample** | **# Total reads** | **# Quality-trimmed reads** | **% Reads mapped to MAGs** |
| --- | --- | --- | --- | --- |
| metagenomic sequencing data | High_BS_d730 | 70,445,180 | 57,779,160 | 77.8 |
|  | High_BS_d1095 | 81,942,612 | 66,805,828 | 74.7 |
|  | Low_BS_d730 | 71,558,844 | 58,479,224 | 76.0 |
|  | Low_BS_d1095 | 57,393,052 | 47,116,400 | 73.2 |
| metatranscriptomic sequencing data | High_BS_RNA_d1095 | 132,243,102 | 98,327,222 | 73.6 |
|  | Low_BS_RNA_d1095 | 162,541,986 | 121,854,526 | 60.6 |

**Table S4. Characteristics of 56 high-quality recovered MAGs with >= 75% completeness and <=10% contamination in both communities.** Assessment of MAGs were performed using checkM, lineage information of each MAG was determined using GTDBTk.

| **Bin name** | **Complet. %** | **Contam. %** | **Genome size (M)** | **# contigs** | **Maximal contig (bp)** | **N50** | **GC (%)** | **# cds** | **Lineage information** |
| --- | --- | --- | --- | --- | --- | --- | --- | --- | --- |
| PLA1 | 96.52 | 0 | 3.23 | 63 | 220,600 | 93,926 | 68.63 | 2,691 | d__Bacteria;p__Planctomycetota;c__Phycisphaerae;o__Phycisphaerales;f__SM1A02;g__;s__ |
| ANME1 | 99.35 | 0.65 | 2.95 | 56 | 286,942 | 60,495 | 42.84 | 3,149 | d__Archaea;p__Halobacterota;c__Methanosarcinia;o__Methanosarcinales;f__Methanoperedenaceae;g__Methanoperedens;s__Methanoperedens nitroreducens |
| AMXB1 | 97.8 | 0.55 | 3.6 | 67 | 182,235 | 80,273 | 42.04 | 3,282 | d__Bacteria;p__Planctomycetota;c__Brocadiae;o__Brocadiales;f__Brocadiaceae;g__Brocadia;s__ |
| DAMOB1 | 94.4 | 2.56 | 3.03 | 49 | 248,628 | 129,037 | 59.8 | 3,009 | d__Bacteria;p__Methylomirabilota;c__Methylomirabilia;o__Methylomirabilales;f__Methylomirabilaceae;g__Methylomirabilis;s__Methylomirabilis sp003104975 |
| CHL5 | 97.27 | 8.18 | 10.41 | 250 | 270,416 | 60,961 | 55.17 | 8,908 | d__Bacteria;p__Chloroflexota;c__Anaerolineae;o__4572-78;f__;g__;s__ |
| AMXK1 | 87.36 | 0.55 | 3.05 | 430 | 33,703 | 8,268 | 40.66 | 2,739 | d__Bacteria;p__Planctomycetota;c__Brocadiae;o__Brocadiales;f__Brocadiaceae;g__Kuenenia;s__Kuenenia stuttgartiensis |
| PRO2 | 88.03 | 4.81 | 3.64 | 290 | 88,482 | 18,228 | 61.1 | 3,735 | d__Bacteria;p__Proteobacteria;c__Gammaproteobacteria;o__Burkholderiales;f__Rhodocyclaceae;g__UTPRO2;s__UTPRO2 sp002840845 |
| ZXB1 | 100 | 0 | 3.78 | 58 | 377,927 | 92,046 | 61.22 | 3,085 | d__Bacteria;p__Zixibacteria;c__MSB-5A5;o__;f__;g__;s__ |
| CHL2 | 75.2 | 9.42 | 4.85 | 653 | 42,400 | 8,728 | 59.9 | 4,783 | d__Bacteria;p__Chloroflexota;c__Anaerolineae;o__SBR1031;f__UBA2029;g__UBA2029;s__ |
| BAC2 | 96.65 | 0 | 3.68 | 65 | 283,483 | 122,147 | 33.23 | 3,054 | d__Bacteria;p__Bacteroidota;c__Ignavibacteria;o__Ignavibacteriales;f__Ignavibacteriaceae;g__Ignavibacterium;s__ |
| CHL1 | 91.82 | 1.27 | 3.21 | 122 | 98,299 | 33,991 | 59.07 | 2,894 | d__Bacteria;p__Chloroflexota;c__Anaerolineae;o__Anaerolineales;f__envOPS12;g__UBA7227;s__UBA7227 sp002473085 |
| PRO3 | 77.59 | 8.62 | 3.65 | 120 | 202,137 | 57,327 | 66.21 | 3,508 | d__Bacteria;p__Proteobacteria;c__Gammaproteobacteria;o__Burkholderiales;f__Rhodocyclaceae;g__UTPRO2;s__ |
| PRO1 | 98.6 | 0.93 | 4.83 | 74 | 322,579 | 148,425 | 69.43 | 4,513 | d__Bacteria;p__Proteobacteria;c__Gammaproteobacteria;o__Burkholderiales;f__Burkholderiaceae;g__JOSHI-001;s__ |
| BAC1 | 99.44 | 0 | 3.66 | 34 | 311,297 | 152,382 | 34.57 | 3,141 | d__Bacteria;p__Bacteroidota;c__Ignavibacteria;o__Ignavibacteriales;f__Ignavibacteriaceae;g__BMS3ABIN03;s__ |
| CHL8 | 95.45 | 1.82 | 6.68 | 173 | 508,039 | 73,711 | 63.84 | 5,312 | d__Bacteria;p__Chloroflexota;c__Anaerolineae;o__SBR1031;f__UBA2029;g__;s__ |
| ARM1 | 93.98 | 0.93 | 2.81 | 35 | 320,577 | 131,807 | 60.98 | 2,575 | d__Bacteria;p__Armatimonadota;c__Fimbriimonadia;o__Fimbriimonadales;f__Fimbriimonadaceae;g__OLB18;s__OLB18 sp001567425 |
| CHL4 | 92.55 | 1.09 | 4.04 | 65 | 207,595 | 94,568 | 53 | 3,783 | d__Bacteria;p__Chloroflexota;c__Anaerolineae;o__Anaerolineales;f__envOPS12;g__UBA12294;s__ |
| PLA2 | 97.73 | 0 | 5.88 | 179 | 178,572 | 58,317 | 70.96 | 4,661 | d__Bacteria;p__Planctomycetota;c__UBA1135;o__UBA2386;f__UBA2386;g__UBA2386;s__ |
| ACI1 | 90.6 | 7.69 | 6.19 | 230 | 169,762 | 39,568 | 73.11 | 5,102 | d__Bacteria;p__Acidobacteriota;c__Vicinamibacteria;o__Fen-336;f__;g__;s__ |
| PRO5 | 83.01 | 2.05 | 3.06 | 82 | 247,187 | 62,328 | 66.81 | 2,763 | d__Bacteria;p__Proteobacteria;c__Gammaproteobacteria;o__GCA-2729495;f__GCA-2729495;g__;s__ |
| VER2 | 82.09 | 4.05 | 6.25 | 658 | 68,236 | 13,135 | 66.22 | 4,954 | d__Bacteria;p__Verrucomicrobiota;c__Verrucomicrobiae;o__;f__;g__;s__ |
| PRO16 | 94.83 | 8.62 | 3.01 | 145 | 147,041 | 27,596 | 62.52 | 2,985 | d__Bacteria;p__Proteobacteria;c__Gammaproteobacteria;o__Burkholderiales;f__Hydrogenophilaceae;g__Thiobacillus;s__Thiobacillus sp001899775 |
| PLA5 | 91.98 | 4.55 | 4.38 | 89 | 332,083 | 97,436 | 65.09 | 3,603 | d__Bacteria;p__Planctomycetota;c__UBA8742;o__UBA2392;f__UBA2392;g__;s__ |
| AMXB2 | 92.31 | 2.75 | 4 | 174 | 152,077 | 40,921 | 41.62 | 3,740 | d__Bacteria;p__Planctomycetota;c__Brocadiae;o__Brocadiales;f__Brocadiaceae;g__Brocadia;s__ |
| DAMOB2 | 95.39 | 2.56 | 2.64 | 42 | 257,088 | 126,436 | 59.13 | 2,647 | d__Bacteria;p__Methylomirabilota;c__Methylomirabilia;o__Methylomirabilales;f__Methylomirabilaceae;g__Methylomirabilis;s__Methylomirabilis sp003105305 |
| PRO4 | 95.71 | 3.71 | 5.89 | 237 | 118,464 | 34,801 | 69.6 | 5,606 | d__Bacteria;p__Proteobacteria;c__Alphaproteobacteria;o__Dongiales;f__Dongiaceae;g__;s__ |
| PLA4 | 94.32 | 0 | 4.74 | 210 | 112,048 | 42,328 | 66.32 | 4,104 | d__Bacteria;p__Planctomycetota;c__Phycisphaerae;o__Phycisphaerales;f__SM1A02;g__UBA5793;s__ |
| CHL3 | 95.15 | 4.91 | 5.8 | 228 | 105,934 | 40,071 | 56.68 | 5,187 | d__Bacteria;p__Chloroflexota;c__Anaerolineae;o__UBA4142;f__UBA4142;g__;s__ |
| PRO13 | 75.76 | 4.24 | 3.19 | 123 | 112,489 | 40,775 | 69.01 | 2,982 | d__Bacteria;p__Proteobacteria;c__Gammaproteobacteria;o__Burkholderiales;f__Burkholderiaceae;g__SCN-69-89;s__ |
| PRO11 | 98.15 | 3.74 | 4.01 | 70 | 224,227 | 102,522 | 65.57 | 3,881 | d__Bacteria;p__Proteobacteria;c__Alphaproteobacteria;o__Rhizobiales;f__Xanthobacteraceae;g__Pseudorhodoplanes;s__ |
| ACI2 | 88.5 | 1.8 | 5.42 | 541 | 63,749 | 12,326 | 72.9 | 4,553 | d__Bacteria;p__Acidobacteriota;c__Thermoanaerobaculia;o__UBA5066;f__UBA5066;g__;s__ |
| PLA3 | 95.1 | 2.6 | 6.93 | 324 | 137,658 | 34,206 | 66.34 | 5,214 | d__Bacteria;p__Planctomycetota;c__SZUA-567;o__;f__;g__;s__ |
| ACI3 | 98.29 | 4.44 | 5.34 | 134 | 194,530 | 81,441 | 61 | 4,473 | d__Bacteria;p__Acidobacteriota;c__Acidobacteriae;o__Bryobacterales;f__;g__;s__ |
| PLA3 | 75.7 | 0 | 9.32 | 1630 | 29,888 | 6,402 | 62.05 | 7,706 | d__Bacteria;p__Planctomycetota;c__Planctomycetes;o__Pirellulales;f__Pirellulaceae;g__;s__ |
| VER1 | 84.5 | 6.01 | 8.41 | 1089 | 37,320 | 9,740 | 62.28 | 6,938 | d__Bacteria;p__Verrucomicrobiota;c__Verrucomicrobiae;o__Pedosphaerales;f__AV2;g__;s__ |
| CHL6 | 85 | 3.18 | 4.15 | 347 | 86,345 | 15,358 | 51.54 | 4,018 | d__Bacteria;p__Chloroflexota;c__Anaerolineae;o__Anaerolineales;f__envOPS12;g__OLB14;s__ |
| PRO15 | 90.68 | 0.52 | 2.93 | 45 | 215,594 | 101,158 | 69.45 | 2,525 | d__Bacteria;p__Proteobacteria;c__Gammaproteobacteria;o__Xanthomonadales;f__Rhodanobacteraceae;g__Dokdonella;s__ |
| CYA1 | 91.45 | 3.42 | 6.34 | 270 | 179,422 | 37,025 | 51.22 | 5,550 | d__Bacteria;p__Cyanobacteria;c__Vampirovibrionia;o__Obscuribacterales;f__Obscuribacteraceae;g__QKMZ01;s__ |
| CHL7 | 90.91 | 1.09 | 3.81 | 100 | 231,448 | 63,620 | 54.61 | 3,608 | d__Bacteria;p__Chloroflexota;c__Anaerolineae;o__Anaerolineales;f__envOPS12;g__UBA12294;s__ |
| PRO9 | 86.26 | 5.26 | 4.27 | 524 | 54,407 | 10,485 | 67.28 | 3,891 | d__Bacteria;p__Proteobacteria;c__Gammaproteobacteria;o__Ga0077536;f__;g__;s__ |
| OLB1 | 94.35 | 3.23 | 3.89 | 445 | 51,202 | 10,796 | 54.5 | 3,273 | d__Bacteria;p__OLB16;c__OLB16;o__OLB16;f__OLB16;g__OLB16;s__OLB16 sp001567115 |
| CYA2 | 95.73 | 4.42 | 6.03 | 225 | 153,945 | 37,865 | 56.97 | 5,316 | d__Bacteria;p__Cyanobacteria;c__Vampirovibrionia;o__Obscuribacterales;f__;g__;s__ |
| DAMUBA1 | 94.84 | 2.53 | 3.33 | 197 | 83,586 | 22,821 | 63.34 | 2,775 | d__Bacteria;p__UBA10199;c__UBA10199;o__;f__;g__;s__ |
| MYX1 | 78.12 | 1.94 | 4.53 | 565 | 39,035 | 9,931 | 68.96 | 4,056 | d__Bacteria;p__Myxococcota;c__UBA9160;o__UBA9160;f__UBA9160;g__;s__ |
| PRO7 | 99.34 | 0.16 | 3.46 | 55 | 345,590 | 10,0613 | 62.73 | 3,380 | d__Bacteria;p__Proteobacteria;c__Alphaproteobacteria;o__Rhizobiales;f__Beijerinckiaceae;g__Methylocystis;s__ |
| PRO8 | 84.72 | 7.76 | 4.95 | 727 | 34,928 | 7,965 | 63.62 | 4,942 | d__Bacteria;p__Proteobacteria;c__Gammaproteobacteria;o__Burkholderiales;f__SG8-39;g__2-12-FULL-64-23;s__ |
| PRO6 | 95.86 | 3.21 | 4.36 | 338 | 87,665 | 19,698 | 53.03 | 4,086 | d__Bacteria;p__Proteobacteria;c__Gammaproteobacteria;o__Methylococcales;f__Methylomonadaceae;g__Methylomonas;s__ |
| CHL9 | 76.38 | 2.73 | 4.09 | 685 | 48,499 | 6,440 | 53.58 | 3,821 | d__Bacteria;p__Chloroflexota;c__Anaerolineae;o__Promineofilales;f__Promineofilaceae;g__GCA-2746795;s__ |
| PRO12 | 77.5 | 2.96 | 2.72 | 371 | 43,887 | 9,407 | 68.64 | 2,593 | d__Bacteria;p__Proteobacteria;c__Gammaproteobacteria;o__GCA-2729495;f__GCA-2729495;g__;s__ |
| DES1 | 84.62 | 3.91 | 4.24 | 720 | 36,024 | 6,648 | 67.79 | 3,708 | d__Bacteria;p__Desulfobacterota;c__Desulfobacteria;o__Desulfobacterales;f__Desulfosarcinaceae_B;g__UBA5852;s__ |
| PAT1 | 76.24 | 0 | 1.1 | 8 | 462,724 | 218,937 | 59.28 | 1,098 | d__Bacteria;p__Patescibacteria;c__ABY1;o__SG8-24;f__2-12-FULL-60-25;g__21-14-all-47-17;s__ |
| ACT1 | 91.2 | 2.41 | 4.63 | 562 | 45,095 | 10,043 | 67.11 | 4,531 | d__Bacteria;p__Actinobacteriota;c__Actinobacteria;o__Mycobacteriales;f__Mycobacteriaceae;g__Mycolicibacter;s__ |
| NIT1 | 84.9 | 4.77 | 4.24 | 388 | 61,950 | 15,116 | 59.19 | 4,193 | d__Bacteria;p__Nitrospirota;c__Nitrospiria;o__Nitrospirales;f__Nitrospiraceae;g__Nitrospira_A;s__Nitrospira_A sp003456605 |
| PRO10 | 90.69 | 2.94 | 2.35 | 259 | 55,147 | 11,521 | 62.67 | 2,262 | d__Bacteria;p__Proteobacteria;c__Gammaproteobacteria;o__Burkholderiales;f__;g__;s__ |
| FEN1 | 81.62 | 0.61 | 3.11 | 422 | 54,342 | 8,794 | 61.57 | 2,894 | d__Bacteria;p__FEN-1099;c__FEN-1099;o__;f__;g__;s__ |
| PRO14 | 79.56 | 1.35 | 2.17 | 342 | 26,309 | 7,743 | 66.36 | 2,183 | d__Bacteria;p__Proteobacteria;c__Alphaproteobacteria;o__Caulobacterales;f__Caulobacteraceae;g__Brevundimonas;s__ |

**Table S5. Relative abundance of MAGs.** Relative abundance of each MAG was calculated as mapped quality-trimmed reads divided by total quality-trimmed reads of each sample.

| **MAG name** | **High_BS_d730 (%)** | **High_BS_d1095 (%)** | **Low_BS_d730(%)** | **Low_BS_d1095(%)** |
| --- | --- | --- | --- | --- |
| PLA1 | 21.78 | 15.43 | 9.83 | 6.67 |
| ANME1 | 19.03 | 17.81 | 0.73 | 0.38 |
| AMXB1 | 2.05 | 1.28 | 14.89 | 14.19 |
| DAMOB1 | 0.22 | 0.15 | 13.98 | 10.84 |
| CHL5 | 2.75 | 3.83 | 0.88 | 0.88 |
| AMXK1 | 3.53 | 3.63 | 0.64 | 0.35 |
| PRO2 | 2.49 | 1.81 | 1.98 | 1.71 |
| ZXB1 | 0.01 | 0.01 | 2.56 | 4.90 |
| CHL2 | 1.60 | 2.60 | 1.46 | 1.80 |
| BAC2 | 2.87 | 3.08 | 0.94 | 0.40 |
| CHL1 | 2.30 | 1.00 | 2.04 | 1.84 |
| PRO3 | 1.73 | 1.63 | 0.99 | 1.15 |
| PRO1 | 0.51 | 0.59 | 2.06 | 2.00 |
| BAC1 | 0.36 | 0.24 | 2.87 | 1.31 |
| CHL8 | 1.57 | 2.06 | 0.33 | 0.38 |
| ARM1 | 0.91 | 1.40 | 1.04 | 0.70 |
| CHL4 | 0.03 | 0.01 | 2.55 | 1.06 |
| PLA2 | 0.03 | 0.05 | 1.04 | 2.44 |
| ACI1 | 0.09 | 0.09 | 1.26 | 1.96 |
| PRO5 | 0.82 | 1.11 | 0.65 | 0.75 |
| VER2 | 0.99 | 1.95 | 0.06 | 0.05 |
| PRO16 | 1.21 | 1.71 | 0.07 | 0.06 |
| PLA5 | 1.39 | 1.32 | 0.18 | 0.13 |
| AMXB2 | 1.10 | 1.45 | 0.23 | 0.21 |
| DAMOB2 | 0.01 | 0.01 | 0.90 | 1.69 |
| PRO4 | 0.51 | 0.86 | 0.38 | 0.77 |
| PLA4 | 0.56 | 0.97 | 0.29 | 0.55 |
| CHL3 | 0.18 | 0.37 | 0.69 | 1.10 |
| PRO13 | 0.75 | 0.94 | 0.31 | 0.27 |
| PRO11 | 0.73 | 0.72 | 0.39 | 0.35 |
| ACI2 | 0.06 | 0.05 | 1.02 | 1.00 |
| PLA3 | 0.02 | 0.04 | 0.58 | 1.42 |
| ACI3 | 0.61 | 0.75 | 0.26 | 0.28 |
| PLA3 | 0.18 | 0.13 | 0.60 | 0.94 |
| VER1 | 0.13 | 0.18 | 0.49 | 0.97 |
| CHL6 | 0.26 | 0.46 | 0.38 | 0.65 |
| PRO15 | 0.98 | 0.39 | 0.09 | 0.07 |
| CYA1 | 0.01 | 0.00 | 0.75 | 0.76 |
| CHL7 | 0.04 | 0.02 | 0.87 | 0.53 |
| PRO9 | 0.22 | 0.35 | 0.34 | 0.49 |
| OLB1 | 0.27 | 0.32 | 0.46 | 0.33 |
| CYA2 | 0.50 | 0.87 | 0.00 | 0.00 |
| DAMUBA1 | 0.25 | 0.27 | 0.59 | 0.25 |
| MYX1 | 0.34 | 0.39 | 0.28 | 0.25 |
| PRO7 | 0.02 | 0.02 | 0.58 | 0.59 |
| PRO8 | 0.09 | 0.09 | 0.32 | 0.57 |
| PRO6 | 0.01 | 0.01 | 0.35 | 0.66 |
| CHL9 | 0.26 | 0.29 | 0.31 | 0.13 |
| PRO12 | 0.25 | 0.22 | 0.23 | 0.27 |
| DES1 | 0.41 | 0.51 | 0.02 | 0.01 |
| PAT1 | 0.33 | 0.53 | 0.03 | 0.02 |
| ACT1 | 0.02 | 0.02 | 0.28 | 0.55 |
| NIT1 | 0.01 | 0.01 | 0.27 | 0.52 |
| PRO10 | 0.02 | 0.02 | 0.18 | 0.36 |
| FEN1 | 0.13 | 0.28 | 0.01 | 0.01 |
| PRO14 | 0.01 | 0.01 | 0.15 | 0.19 |
| Total | 77.57 | 74.32 | 75.64 | 72.76 |

**Table S6. TPM (transcripts per million) value of each MAGs.** The MAG TPM was calculated as sum of TPM of predicted CDS in the MAG. MAGs with expression > 1% in at least one bioreactor community are shown.

| MAG name | High_BS_RNA | Low_BS_RNA |
| --- | --- | --- |
| DAMOA1 | 60.44 | 0.04 |
| DAMOB1 | 0.01 | 3.29 |
| DAMOB2 | 0 | 2.38 |
| AMXK1 | 9.67 | 1.40 |
| AMXB1 | 2.95 | 61.67 |
| AMXB2 | 5.03 | 1.24 |
| IGN1 | 5.07 | 0.09 |
| VER1 | 0.05 | 7.01 |
| CHL3 | 0.01 | 4.13 |
| PRO7 | 0 | 1.36 |

Legend of supplementary data sets.

**Supplementary Dataset 1**. Genes annotated as peptidases of each MAG and the expression comparing to the median gene expression value are listed in sheet “Peptidases”. Expression of pathways compared to median gene expression for amino acids biosynthesis and degradation are listed in sheet “Amino acids metabolism”. Expression of transporters, that involved in amino acids, peptides, and proteins, comparing to median gene expression are listed in sheet “transporters-tpm”, gene counts of the transporters are listed in sheet “transporters-gene_counts”.

**Supplementary Dataset 2.** Gene annotation, TPM (Transcripts per million) values of active MAGs in both communities are listed in the sheets. Each sheet represents one genome.

**Supplementary Dataset 3.** Expression of pathways of active MAGs are listed. Each sheet represents one genome.

**References**

1 Bolger, A. M., Lohse, M. & Usadel, B. Trimmomatic: a flexible trimmer for Illumina sequence data. *Bioinformatics*. 2014; 30: 2114-2120.

2 Magoc, T. & Salzberg, S. L. FLASH: fast length adjustment of short reads to improve genome assemblies. *Bioinformatics*. 2011; 27: 2957-2963.

3 Edgar, R. C. UPARSE: highly accurate OTU sequences from microbial amplicon reads. *Nat Methods*. 2013; 10: 996-998.

4 Edgar, R. C. Search and clustering orders of magnitude faster than BLAST. *Bioinformatics*. 2010; 26: 2460-2461.

5 Caporaso, J. G. *et al.* QIIME allows analysis of high-throughput community sequencing data. *Nat Methods*. 2010; 7: 335-336.

6 Wang, Q., Garrity, G. M., Tiedje, J. M. & Cole, J. R. Naive Bayesian classifier for rapid assignment of rRNA sequences into the new bacterial taxonomy. *Appl Environ Microbiol*. 2007; 73: 5261-5267.

7 Quast, C. *et al.* The SILVA ribosomal RNA gene database project: improved data processing and web-based tools. *Nucleic Acids Res*. 2013; 41: D590-596.

8 Seemann, T. Prokka: rapid prokaryotic genome annotation. *Bioinformatics*. 2014; 30: 2068-2069.

9 Ogata, H. *et al.* KEGG: Kyoto Encyclopedia of Genes and Genomes. *Nucleic Acids Res*. 1999; 27: 29-34.

10 Huerta-Cepas, J. *et al.* eggNOG 5.0: a hierarchical, functionally and phylogenetically annotated orthology resource based on 5090 organisms and 2502 viruses. *Nucleic Acids Res*. 2019; 47: D309-D314.

11 UniProt, C. UniProt: a worldwide hub of protein knowledge. *Nucleic Acids Res*. 2019; 47: D506-D515.

12 Aramaki, T. *et al.* KofamKOALA: KEGG ortholog assignment based on profile HMM and adaptive score threshold. *Bioinformatics*. 2019.

13 Huerta-Cepas, J. *et al.* Fast Genome-Wide Functional Annotation through Orthology Assignment by eggNOG-Mapper. *Molecular Biology and Evolution*. 2017; 34: 2115-2122.

14 Buchfink, B., Xie, C. & Huson, D. H. Fast and sensitive protein alignment using DIAMOND. *Nature Methods*. 2015; 12: 59-60.

15 Saier, M. H., Jr. *et al.* The Transporter Classification Database (TCDB): recent advances. *Nucleic Acids Res*. 2016; 44: D372-379.

16 Caspi, R. *et al.* The MetaCyc database of metabolic pathways and enzymes - a 2019 update. *Nucleic Acids Res*. 2020; 48: D445-D453.

17 Hyatt, D. *et al.* Prodigal: prokaryotic gene recognition and translation initiation site identification. *BMC Bioinformatics*. 2010; 11: 119.

18 Price, M. N., Dehal, P. S. & Arkin, A. P. FastTree 2--approximately maximum-likelihood trees for large alignments. *PLoS One*. 2010; 5: e9490.

19 Letunic, I. & Bork, P. Interactive Tree Of Life (iTOL) v5: an online tool for phylogenetic tree display and annotation. *Nucleic Acids Res*. 2021; 49: W293-W296.

20 Olm, M. R., Brown, C. T., Brooks, B. & Banfield, J. F. dRep: a tool for fast and accurate genomic comparisons that enables improved genome recovery from metagenomes through de-replication. *ISME Journal*. 2017; 11: 2864-2868.

21 Yu, C. S., Lin, C. J. & Hwang, J. K. Predicting subcellular localization of proteins for Gram-negative bacteria by support vector machines based on n-peptide compositions. *Protein Sci*. 2004; 13: 1402-1406.

22 Akashi, H. & Gojobori, T. Metabolic efficiency and amino acid composition in the proteomes of Escherichia coli and Bacillus subtilis. *Proc Natl Acad Sci U S A*. 2002; 99: 3695-3700.
